# Supplementary material for: The Evolution and Characterization of the RNA Interference Pathways in Lophotrochozoa
Source: Genome Biol Evol. 2024 May 7;16(5):evae098. doi: 10.1093/gbe/evae098 (PMC11114477; doi:10.1093/gbe/evae098)
Supplement: evae098_Supplementary_Data [file evae098_supplementary_data.zip › Supplementary_materials.zip!/selected_miRNAs/NoGe_selected_miRNAs/mirdeep_pdf_files/NMRB01001155.1_Notospermus_geniculatus_strain_Ushimado_scaffold1155,_whole_genome_shotgun_sequence_25652.pdf]

Provisional ID	:	NMRB01001155.1_Notospermus_geniculatus_strain_Ushimado_scaffold1155,_whole_genome_shotgun_sequence_25652
Score total	:	846
Score for star read(s)	:	3.9
Score for read counts	:	840.8
Score for mfe	:	1.9
Score for randfold	:	0
Score for cons. seed	:	-0.6
Total read count	:	1661
Mature read count	:	1649
Loop read count	:	0
Star read count	:	12

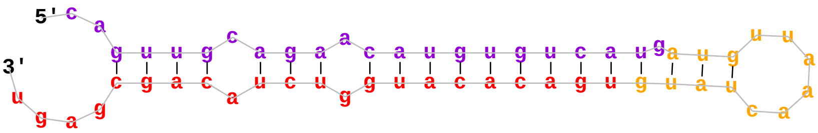

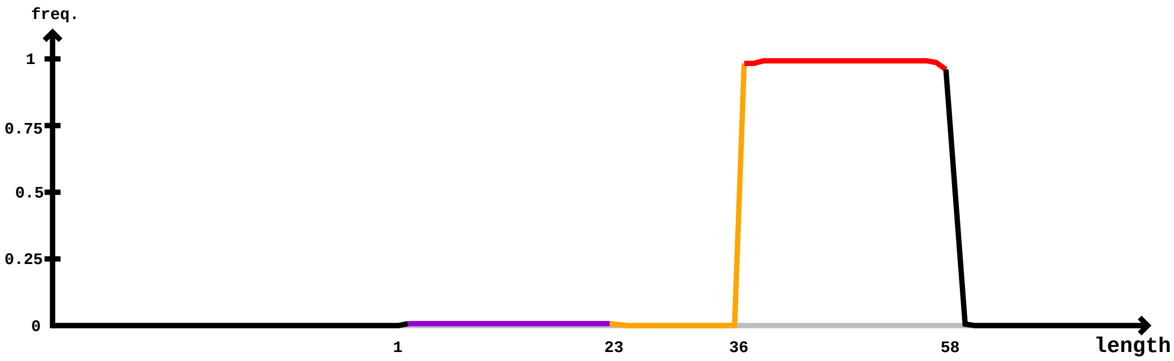

Star Mature

```
5'- uugaugcaugcgguauagauacugucacgacgcaccaguugcagaacaugugucaugauguuaaacuaugugacacauggucuacagcgaguguguugggcaggguuucaac  -3'   obs
    uugaugcaugcgguauagauacugucacgacgcaccaguugcagaacaugugucaugauguuaaacuaugugacacauggucuacagcgaguguguugggcaggguuucaac         exp
    ...((((.....))))((((.(((((.((((((((..((((.(((.((((((((((.(((......))))))))))))).))).)))).)))))))))))))).))))....     reads   mm      sample
    ...................................caguugcagaacaugugucaug.......................................................     1       0       Mm3
    ...................................caguugcagaacaugugucaugU......................................................     1       1       Mm3
    ......................................................................ugacacauggucuacagcgG......................     1       1       Mm3
    ......................................................................ugacacauggucuacagcga......................     1       0       Mm3
    ......................................................................ugacacauggucuacagcgag.....................     4       0       Mm3
    ......................................................................ugacacauggucuacagUgagu....................     2       1       Mm3
    ......................................................................ugacacauggucuacagcgagu....................     106     0       Mm3
    ......................................................................ugacacauggucuacCgcgagu....................     1       1       Mm3
    ......................................................................ugacacauggucuacaAcgagu....................     1       1       Mm3
    ......................................................................ugacacauggucuacagAgagu....................     1       1       Mm3
    ......................................................................ugacacauggucuacagGgagu....................     1       1       Mm3
    ......................................................................ugacacauggucuacagcgaguA...................     1       1       Mm3
    ......................................................................ugacacauggucuacagcgaguU...................     6       1       Mm3
    ........................................................................acacauggucuacagcgagu....................     1       0       Mm3

    .....................................................................Cugacacauggucuacagcgagu....................     1       1       Fm3
    ......................................................................ugacacauggucuacagcga......................     1       0       Fm3
    ......................................................................ugacacauggucuacagcgU......................     2       1       Fm3
    ......................................................................ugacacauggucuacagGga......................     1       1       Fm3
    ......................................................................ugacacauggucuacaCcga......................     1       1       Fm3
    ......................................................................ugacacauggucuacagcgag.....................     17      0       Fm3
    ......................................................................ugacacauggucuacagAgag.....................     2       1       Fm3
    ......................................................................ugacacauggucuacaAcgagu....................     3       1       Fm3
    ......................................................................ugacacauggucuacagcUagu....................     1       1       Fm3
    ......................................................................ugacacauggucuacagAgagu....................     8       1       Fm3
    ......................................................................ugacacauggucuacagcgagA....................     1       1       Fm3
    ......................................................................ugacacauggucuacagGgagu....................     2       1       Fm3
    ......................................................................ugacacauggucuacagcgagu....................     758     0       Fm3
    ......................................................................ugacacauggucuacagUgagu....................     5       1       Fm3
    ......................................................................uCacacauggucuacagcgagu....................     1       1       Fm3
    ......................................................................ugacacauggCcuacagcgagu....................     1       1       Fm3
    ......................................................................ugacacauggucuacaUcgagu....................     1       1       Fm3
    ......................................................................ugacacauggucuacagcgaguU...................     32      1       Fm3
```

```
                                   Star                             Mature
uugaugcaugcgguauagauacugucacgacgcaccaguugcagaacaugugucaugauguuaaacuaugugacacauggucuacagcgaguguguugggcaggguuucaac

...................................caguugcagaacaugugucaug.......................................................    4     0    Mm2
......................................................................ugacacauggucuacagcgag.....................    2     0    Mm2
......................................................................ugaAacauggucuacagcgagu....................    1     1    Mm2
......................................................................ugacacauggucuacaAcgagu....................    1     1    Mm2
......................................................................ugacacauggucuacagcgagu....................    292   0    Mm2
......................................................................ugacacauggucuacagGgagu....................    1     1    Mm2
......................................................................ugacacauUgucuacagcgagu....................    1     1    Mm2
......................................................................ugacaUauggucuacagcgagu....................    1     1    Mm2
......................................................................ugacacauggucuacagAgagu....................    3     1    Mm2
......................................................................ugacacauggucuacagcgaguU...................    10    1    Mm2
......................................................................ugacacauggucuacagcgaguA...................    1     1    Mm2
......................................................................ugacacauggucuacagcgaguUu..................    6     1    Mm2
......................................................................ugacacauggucuacagcgaguAu..................    2     1    Mm2
........................................................................acacauggucuacagcgaguUu..................    1     1    Mm2

......................................................................ugacacauggucuacagcgag.....................    16    0    Mm1
......................................................................ugacacauggucuacaAcgagu....................    1     1    Mm1
......................................................................ugacacauggucuacagAgagu....................    1     1    Mm1
......................................................................ugacacauggucuacagcgagu....................    50    0    Mm1
......................................................................ugacacauggucuacagcgaguU...................    7     1    Mm1
........................................................................acacauggucuacagcgagu....................    13    0    Mm1
........................................................................acacauggucuacagcgGgu....................    1     1    Mm1

......................................................................ugacacauggucuacagcga......................    4     0    Fm1
......................................................................ugacacauggucuacagcgag.....................    2     0    Fm1
......................................................................ugacacauggucuacagUgagu....................    1     1    Fm1
......................................................................ugacacauggucuacagcgGgu....................    1     1    Fm1
......................................................................ugacacauggucuacagGgagu....................    1     1    Fm1
......................................................................ugacacauggucuacagcgagu....................    159   0    Fm1
......................................................................ugacacauggucuacagcgagG....................    1     1    Fm1
......................................................................ugacacauggucuacagcgaguU...................    2     1    Fm1

...................................caguugcagaacauguCucaug.......................................................    1     1    Fm2
...................................caguugcagaacaugugucaug.......................................................    5     0    Fm2
......................................................................ugacacauggucuacagcgagC....................    1     1    Fm2
......................................................................ugacacauggucuacagcgagu....................    99    0    Fm2
......................................................................ugacacauggucuacagGgagu....................    1     1    Fm2
......................................................................ugacacauggucuacagUgagu....................    1     1    Fm2
......................................................................ugacacauUgucuacagcgagu....................    1     1    Fm2
```
